# CD3ε Expression Defines Functionally Distinct Subsets of Vδ1 T Cells in Patients With Human Immunodeficiency Virus Infection

**DOI:** 10.3389/fimmu.2018.00940

**Published:** 2018-05-02

**Authors:** Pádraic J. Dunne, Christina O. Maher, Michael Freeley, Katie Dunne, Andreea Petrasca, Judy Orikiiriza, Margaret R. Dunne, Derval Reidy, Siobhan O’Dea, Aisling Loy, Jim Woo, Aideen Long, Thomas R. Rogers, Fiona Mulcahy, Derek G. Doherty

**Affiliations:** ^1^Discipline of Immunology, School of Medicine, Trinity Translational Medicine Institute, Trinity College Dublin, Dublin, Ireland; ^2^Discipline of Clinical Medicine, School of Medicine, Trinity Translational Medicine Institute, Trinity College Dublin, Dublin, Ireland; ^3^Discipline of Clinical Microbiology, School of Medicine, Trinity Translational Medicine Institute, Trinity College Dublin, Dublin, Ireland; ^4^Genitourinary Infectious Diseases Department, St. James’s Hospital, Dublin, Ireland

**Keywords:** human immunodeficiency virus, Vδ1 T cells, CD3ε, interleukin-17, programmed death-1, flow cytometry

## Abstract

Human γδ T cells expressing the Vδ1 T cell receptor (TCR) recognize self and microbial antigens and stress-inducible molecules in a major histocompatibility complex-unrestricted manner and are an important source of innate interleukin (IL)-17. Vδ1 T cells are expanded in the circulation and intestines of patients with human immunodeficiency virus (HIV) infection. In this study, we show that patients with HIV have elevated frequencies, but not absolute numbers, of circulating Vδ1 T cells compared to control subjects. This increase was most striking in the patients with *Candida albicans* co-infection. Using flow cytometry and confocal microscopy, we identify two populations of Vδ1 T cells, based on low and high expression of the ε chain of the CD3 protein complex responsible for transducing TCR-mediated signals (denoted CD3ε^lo^ and CD3ε^hi^ Vδ1 T cells). Both Vδ1 T cell populations expressed the CD3 ζ-chain, also used for TCR signaling. Using lines of Vδ1 T cells generated from healthy donors, we show that CD3ε can be transiently downregulated by activation but that its expression is restored over time in culture in the presence of exogenous IL-2. Compared to CD3ε^hi^ Vδ1 T cells, CD3ε^lo^ Vδ1 T cells more frequently expressed terminally differentiated phenotypes and the negative regulator of T cell activation, programmed death-1 (PD-1), but not lymphocyte-activation gene 3, and upon stimulation *in vitro*, only the CD3ε^hi^ subset of Vδ1 T cells, produced IL-17. Thus, while HIV can infect and kill IL-17-producing CD4^+^ T cells, Vδ1 T cells are another source of IL-17, but many of them exist in a state of exhaustion, mediated either by the induction of PD-1 or by downregulation of CD3ε expression.

## Introduction

T cells expressing the γδ T cell receptor (TCR) represent a minor population of lymphocytes that expands in blood and peripheral tissues upon exposure to bacteria (1, 2), fungi (3), yeast (4, 5), and viruses (6–8). γδ TCRs bind non-peptide antigens in a major histocompatibility complex (MHC) unrestricted manner, leading to phosphorylation of immunoreceptor tyrosine-based activation motifs (ITAM) on the CD3 γ, δ, ε, ζ, and sometimes FcRγ proteins (9, 10). They respond rapidly by killing target cells, releasing cytokines, and providing ligands that mediate the activation and differentiation of other cells of the immune system (11, 12, 13).

Human γδ T cells comprise three predominant cell populations (Vδ1, Vγ9Vδ2, and Vδ3) based upon differences in the δ chain of the TCR (14, 15). Vδ1 TCRs are diverse and can recognize the stress-inducible proteins MICA and MICB, which are expressed by some tumor and virus-infected cells (16), glycolipid antigens presented by CD1c (17) and CD1d (18, 19) and the algal protein phycoerythrin (20). In addition to the TCR, Vδ1 T cells can be activated *via* ligation of other stimulatory receptors, including NKG2C, NKG2D, NKp30, toll-like receptors, and the β-glucan receptor, dectin 1 (5, 21–24). Upon activation, Vδ1 T cells proliferate, release cytokines, such as interferon-γ (IFN-γ), tumor necrosis factor-α, and interleukin-17 (IL-17), chemokines, such as CCL3, CCL4, and CCL5, and they can kill CD4^+^ T cells *in vitro* (4, 21, 23, 25–27).

Vδ1 T cells are found at higher frequencies in the blood, intestinal mucosa, and bronchoalveolar fluid of patients with human immunodeficiency virus (HIV) compared with healthy subjects (28, 29, 30, 31, 32, 33). We have examined the frequencies, phenotypes, and functions of circulating Vδ1 T cells in a cohort of untreated and antiretroviral therapy (ART)-treated patients with HIV and healthy control subjects. We find that percentage frequencies, but not absolute numbers of Vδ1 T cell are higher in the untreated patients compared to ART-treated patients and control subjects. We also have identified two subsets of Vδ1 T cells based on low and high levels of expression of the CD3ε polypeptide, denoted CD3ε^lo^ and CD3ε^hi^ Vδ1 T cells. Both were expanded in patients with HIV and, in particular, in the patients with *Candida albicans* co-infection. Phenotypic and functional analysis of these Vδ1 T cell subsets indicated that the CD3ε^lo^ cells frequently express terminally differentiated (TD) and exhausted phenotypes and are unable to produce IL-17. These results suggest that HIV may induce a state of Vδ1 T cell inactivation.

## Materials and Methods

### Study Population

Venous blood was obtained from 36 patients with HIV infection (21 males and 15 females) attending the Genitourinary Infectious Diseases Department at St. James’s Hospital, Dublin. At the time of blood sample collection, 22 patients were receiving ART and 14 were not. The CD4^+^ T cell count ranged from 55 to 1,857 (median 529) cells/μl of blood in the treated patients and 261–1,115 (median 578) cell/μl in the untreated patients. The viral load ranged from <50 to 72,796 (median < 50) copies/ml in the treated patients and <50–51,000 (median 578) copies/ml in the untreated patients. Three patients were positive for hepatitis B virus and three were positive for hepatitis C. As controls, blood samples were obtained from 23 healthy age- and gender-matched control subjects. Ethical approval for this study was obtained from the Joint Research Ethics Committee of St. James’s Hospital and Tallaght Hospitals, Dublin, and all participants gave written, informed consent. Buffy coat packs from healthy blood donors were kindly provided by the Irish Blood Transfusion Service. Whole blood was used for enumerating T cells, as described below. Peripheral blood mononuclear cells (PBMCs) were prepared by density gradient centrifugation over Lymphoprep (Nycomed Pharma, Oslo, Norway) and used immediately in all procedures.

### Antibodies and Flow Cytometry

**Figure 1:**
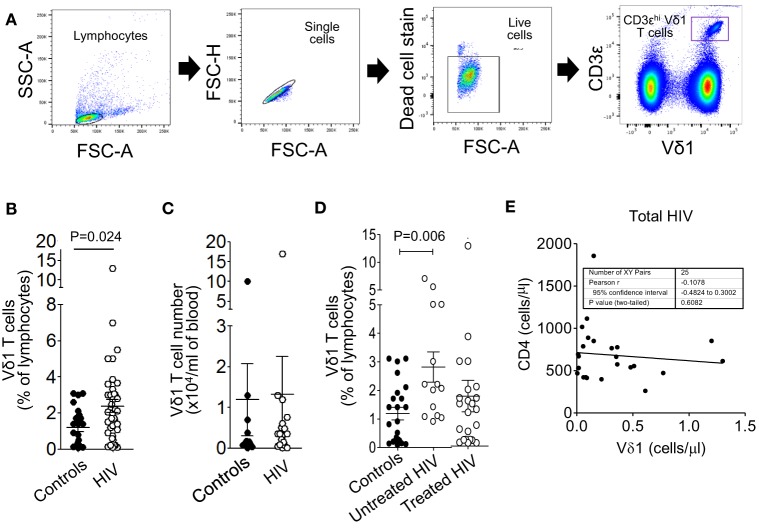
Circulating Vδ1 T cell frequencies but not numbers are higher in patients with untreated human immunodeficiency virus (HIV) infection. Peripheral blood mononuclear cells were prepared from blood samples of 36 patients with HIV infection and 23 healthy donors, stained with monoclonal antibodies specific for CD3 and the Vδ1 T cell receptor and analyzed by flow cytometry. **(A)** Gating strategy for the enumeration of Vδ1 T cells, showing sequential gates on the lymphocytes, single cells, live cells, and Vδ1 T cells. **(B,C)** Scatter plots showing circulating Vδ1 T cell frequencies **(B)** and absolute numbers **(C)**. **(D)** Scatter plots showing Vδ1 T cell frequencies in control subjects (*n* = 23) and HIV patients divided into untreated (*n* = 14) and antiretroviral therapy-treated (*n* = 22) groups. Groups were compared using the Mann–Whitney *U* test. **(E)** Correlation between absolute counts of Vδ1 T cells and total CD4^+^ T cells in the blood of 25 patients with HIV infection.

Fluorochrome-conjugated monoclonal antibodies (mAbs) specific for the human Vδ1 TCR (clone TS-1), CD3ε (clones MEM-1 and HIT-3a), CD3ζ (clone 6B10.2), CD27 (clone 0323), CD45RA (clone HI100), programmed death-1 (PD-1) (clone EH12.1), lymphocyte-activation gene 3 (LAG-3) (clone 11C3C65), and CD31 (clone WM59) were obtained from Thermo Fisher Scientific (Dublin, Ireland), BioLegend (San Diego, CA, USA), and Beckman Coulter (High Wycombe, UK) and used according to the manufacturers’ recommendations. The CD3ε mAb (clone SP4) was kindly provided by Dr. Balbino Alcarón (Severo Ochoa Center for Molecular Biology, Madrid, Spain). Up to 10^6^ PBMC, γδ T cell-enriched PBMC or expanded Vδ1 T cell lines were labeled with mAbs and analyzed using a CyAN ADP (Beckman Coulter) or FACSCanto (Becton Dickinson, Oxford, UK) flow cytometer. Data were analyzed with FlowJo v7.6 (Tree Star, Ashland, OR, USA) software. Single-stained OneComp Beads (Becton Dickinson) were used to set compensation parameters; fluorescence minus one (FMO) and isotype-matched Ab controls were used to set analysis gates. Fixable viability dye (eBioscience) was used to determine cell viability. The gating strategy for enumerating Vδ1 T cells is shown in Figure 1A. Total PBMC were analyzed for the enumeration of γδ T cell subsets. γδ T cell-enriched PBMC, prepared by negative selection using magnetic beads (Miltenyi Biotec, Bergische Gladbach, Germany), were used as a source of Vδ1 T cells for subsequent phenotypic and functional analysis.

### Enumeration of Vδ1 T Cells

Absolute numbers of T cells per μl of blood were determined using Trucount tubes (BD Biosciences) according to the manufacturer’s protocol. The percentages of CD3^+^ cells that expressed Vδ1 TCRs, were determined by flow cytometry, as described above, allowing us to calculate the absolute counts of Vδ1 T cells (per μl of blood).

### Vδ1 T Cell Sorting and Expansion

Lines of Vδ1 T cells were generated from healthy blood donors as described previously (5). Briefly, PBMC were prepared from buffy coat packs and monocytes were isolated by positive selection using CD14 Microbeads (Miltenyi Biotec, Gladbach Bergische, Germany). Monocytes were allowed to differentiate into immature dendritic cells (DCs) by culturing them for 6 days in the presence of granulocyte–monocyte colony-stimulating factor and IL-4 as described (34). Immature DC were plated at densities of 100,000 cells/ml and stimulated overnight with medium only, with heat- or ethanol-killed *C. albicans* (5 × 10^6^ cells/ml) (5). *C. albicans* strain 10231 was obtained from the American Type Culture Collection and cultured for 24 h on malt extract agar. Fungi were cultured for 24 h, isolated, counted, and then inactivated by heating at 96°C for 60 min. Samples were then centrifuged at 5,000 × *g* for 10 min, the supernatants discarded, and the pellets washed with phosphate buffered saline (PBS). Inactivation was confirmed by plating an aliquot onto malt extract agar and incubating for 7 days to check for growth.

Total γδ T cells were enriched from PBMCs using human anti-TCR γ/δ Microbeads (Miltenyi Biotec). γδ-enriched cells (200,000 cells/ml) were cultured in the absence or presence of *C. albicans*- or curdlan-treated DCs at 2:1 ratios in complete serum-free AIM-V medium (AIM-V containing 0.05 mM l-glutamine, 100 U/ml penicillin, 100 U/ml streptomycin, 0.02 M HEPES, 55 µM β-mercaptoethanol, 1× essential amino acids, 1× nonessential amino acids, and 1 mM sodium pyruvate). Co-cultures were challenged with phytohemagglutinin (1 µg/ml; Sigma-Aldrich, Dublin, Ireland) and cultured with rIL-2 (40 U/ml; Miltenyi Biotec), which was added in fresh medium every 2–3 days. Cultures were restimulated every 2 weeks with activated DCs and phytohemagglutinin, which resulted in yields of >10 million Vδ1 T cells by day 28.

### Confocal Microscopy

Expanded Vδ1 T cells were sorted into cells with high and low surface expression of CD3ε using a MoFlo XDP Cell Sorter (Beckman Coulter). The cell populations were subsequently incubated on poly l-lysine-coated 8-well Lab-Tek glass chamber slides (Nunc; Thermo Fisher Scientific) for 30 min at 37°C. The cells were fixed with an equal volume of 8% paraformaldehyde for 15 min at 37°C, permeabilized with 0.3% triton X-100 in PBS for 5 min at room temperature and then blocked with 3% bovine serum albumin in PBS for 30 min at room temperature. The samples were incubated with a fluorescein isothiocyanate (FITC)-conjugated mouse anti-human CD3ε antibody (clone SK7, BioLegend, 1/50 dilution in 3% BSA/PBS) and incubated overnight at 4°C. After two washes in PBS, the slides were counter-stained with Hoechst 33258 (Molecular Probes) for 30 min at room temperature to visualize the nuclei. The slides were then imaged under 63× oil immersion with a Zeiss laser scanning confocal 510 microscope (Carl Zeiss, Hertfordshire, UK). The mean fluorescence intensity (MFI) of CD3 staining and Hoechst staining in individual cells was quantified using Zen 2009 imaging software (Carl Zeiss). The MFI of Hoechst served as an internal reference control between the different populations.

### Analysis of Intracellular Cytokine Production

Interleukin-17 expression by fresh, unexpanded Vδ1 T cells within γδ T cell-enriched PBMCs was examined by flow cytometry after stimulation of the cells for 6 h with medium alone or with 1 ng/ml phorbol myristate acetate (PMA) and 1 µg/ml ionomycin (PMA/I) in the presence of brefeldin A to prevent cytokine release from the cells (5, 34).

### Statistical Analysis

Prism GraphPad software (San Diego, CA, USA) was used for data analysis. Cell frequencies and numbers determined by flow cytometry in subject groups and cytokine levels in treatment groups were compared using the Mann–Whitney *U* test. *P* values <0.05 were considered significant. Correlations were defined using Pearson’s correlation coefficient.

## Results

### Vδ1 T Cell Frequencies but Not Numbers Are Higher in Patients With Untreated HIV Infection

Peripheral blood mononuclear cells were prepared from blood samples of 36 patients with HIV infection and 23 healthy donors, stained with mAbs specific for CD3ε and the Vδ1 TCR and analyzed by flow cytometry (Figure 1A). Figure 1B shows that the frequencies, as percentages of lymphocytes, of Vδ1 T cells were significantly higher in the HIV patient samples. Absolute counts of Vδ1 T cells were not significantly different between patients and controls (Figure 1C), suggesting that the percentage increases in Vδ1 T cells are a result of the depletions of CD4^+^ T cells by HIV. When the patients were divided into untreated (*n* = 14) and ART-experienced (*n* = 22) groups, the frequencies of Vδ1 T cells were found to be higher only in the untreated patients (Figure 1D). Vδ1 T cell numbers did not correlate significantly with total CD4^+^ T cell counts (Figure 1E), suggesting that the increases in Vδ1 T cells in patients with HIV do not simply compensate for the depletions of CD4^+^ T cells. These data confirm and extend previous observations of altered Vδ1 T cell frequencies in patients with HIV.

### Significant Numbers of Vδ1 T Cells Do Not Appear to Express CD3ε

**Figure 2:**
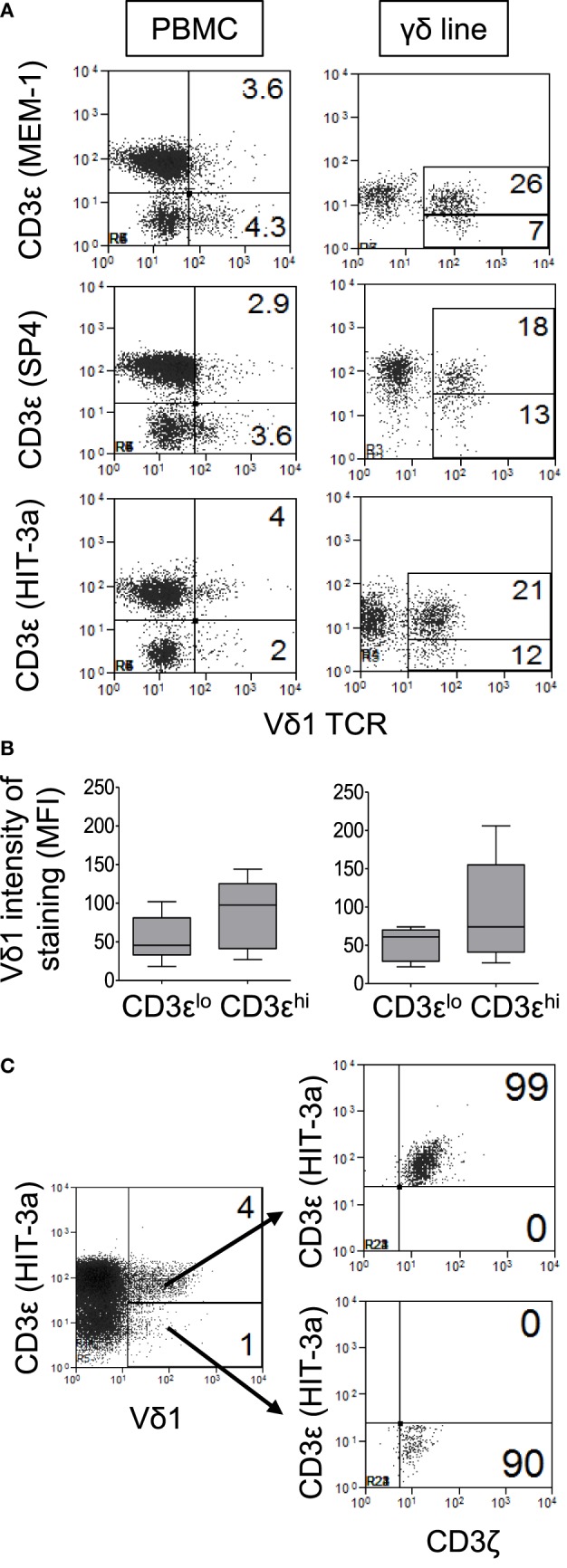
Significant numbers of Vδ1 T cells do not appear to express CD3ε. Peripheral blood mononuclear cell (PBMC) and γδ T cell-enriched PBMC were stained with antibodies specific for Vδ1 and CD3ζ and three different anti-CD3ε monoclonal antibodies (clones MEM-1, SP4, and HIT-3a) in separate tubes and analyzed by flow cytometry. **(A)** Representative flow cytometry dot plots of PBMC (left panels) and expanded Vδ1 T cells (right panels) from a patient with human immunodeficiency virus (HIV) infection showing the expression of CD3ε by Vδ1 T cells. **(B)** Box plots showing mean fluorescence intensities of staining for Vδ1 T cells in CD3ε^lo^ and CD3ε^hi^ Vδ1 T cells from six healthy donors (left) and nine HIV patients (right). **(C)** Representative flow cytometry dot plot showing CD3ζ expression by gated CD3ε^lo^ and CD3ε^hi^ Vδ1 T cells. Results are representative of PBMC or γδ T cell-enriched PBMC from four different donors.

A surprising observation made, while determining the frequencies of Vδ1 T cells in patients and control subjects, was that significant numbers of Vδ1 T cells do not appear to express CD3ε. CD3ε-negative Vδ1 T cells were detected in PBMC and in γδ T cell-enriched PBMC from both patients and control subjects using three different anti-CD3ε mAbs (clones MEM-1, SP4, and HIT-3a) after gating out dead cells, doublets and using FMO controls (Figure 2A). This allowed us to subdivide Vδ1 T cells into two groups on the basis of low and high expression of the TCR co-receptor, denoted CD3ε^lo^ and CD3ε^hi^ Vδ1 T cells, respectively. The levels of Vδ1 TCR expression were slightly higher in CD3ε^hi^ compared to CD3ε^lo^ Vδ1 T cells in both HIV patients and control subjects, although these differences did not reach statistical significance (Figure 2B). Further flow cytometric analysis revealed that both CD3ε^lo^ and CD3ε^hi^ Vδ1 T cells express the CD3ζ polypeptide (Figure 2C).

### CD3ε^lo^ Vδ1 T Cells Express Very Low Levels or No Intracellular CD3ε

**Figure 3:**
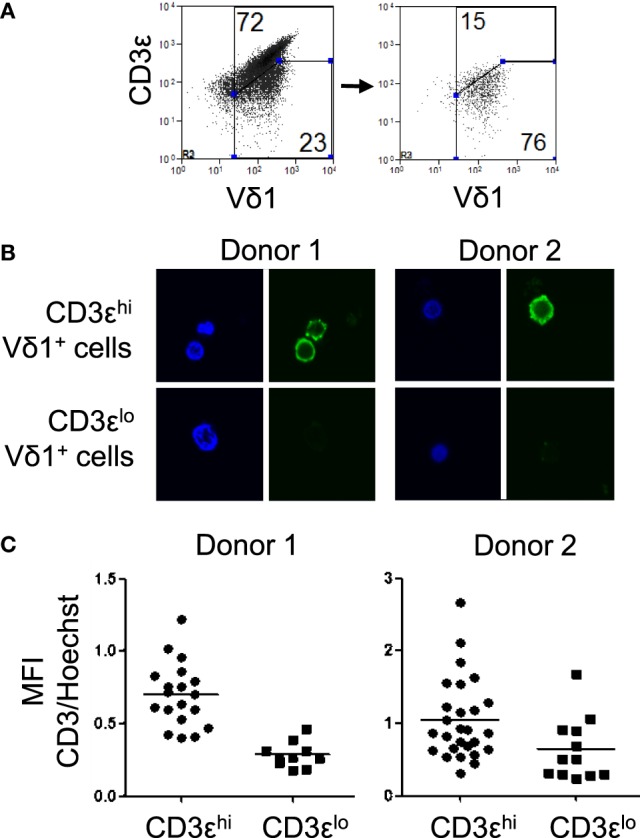
CD3ε^lo^ Vδ1 T cells express very low levels of intracellular CD3ε. Vδ1 T cells were purified from two independent blood donors and sorted using a flow cytometric cell sorter into cells with high and low surface expression of CD3ε. The cell populations were incubated on poly l-lysine-coated glass chamber slides, fixed, permeabilized, and blocked with BSA. The samples were then incubated overnight with a FITC-conjugated mouse anti-human CD3ε antibody, washed twice, and counter-stained with Hoechst 33258 to visualize the nuclei. The slides were then imaged under oil immersion with a laser scanning confocal microscope and mean fluorescence intensity (MFI) was quantified. **(A)** Flow cytometry dot plots showing CD3ε and Vδ1 T cell receptor expression by a line of expanded Vδ1 T cells (left) and sorted CD3ε^lo^ Vδ1 T cells (right). **(B)** Confocal micrographs showing CD3ε (green) and nuclei (blue) in sorted CD3ε^hi^ (top) and CD3ε^lo^ (bottom) Vδ1 T cells from two donors. **(C)** Scatter plots showing the ratios of MFI of CD3 to Hoechst staining in individual cells from the sorted populations of CD3ε^hi^ and CD3ε^lo^ Vδ1 T cells from two donors.

The low levels of CD3ε expression by some Vδ1 T cells may be due to internalization of the CD3ε chain. To investigate if CD3ε^lo^ Vδ1 T cells express intracellular CD3ε, Vδ1 T cells were purified from two healthy donors and sorted by flow cytometry into cells with high and low surface expression of CD3ε (Figure 3A). The cell populations were then bound to slides, fixed, permeabilized, blocked with bovine serum albumin, and stained with a FITC-conjugated mouse anti-human CD3ε antibody and Hoechst 33258. Cells were imaged by confocal microscopy and MFIs were quantified. Figures 3B,C show that CD3ε^hi^ Vδ1 T cells express high levels of cell surface CD3ε and low levels of intracellular CD3ε. By contrast, CD3ε^lo^ Vδ1 T cells express very low levels of cell surface or intracellular CD3ε, indicating that the CD3ε^lo^ Vδ1 T cell phenotype is not the result of internalization CD3ε.

### CD3ε^lo^ and CD3ε^hi^ Vδ1 T Cells Are Both Preserved in Patients With HIV and Especially in Patients With *C. albicans* Co-Infection

**Figure 4:**
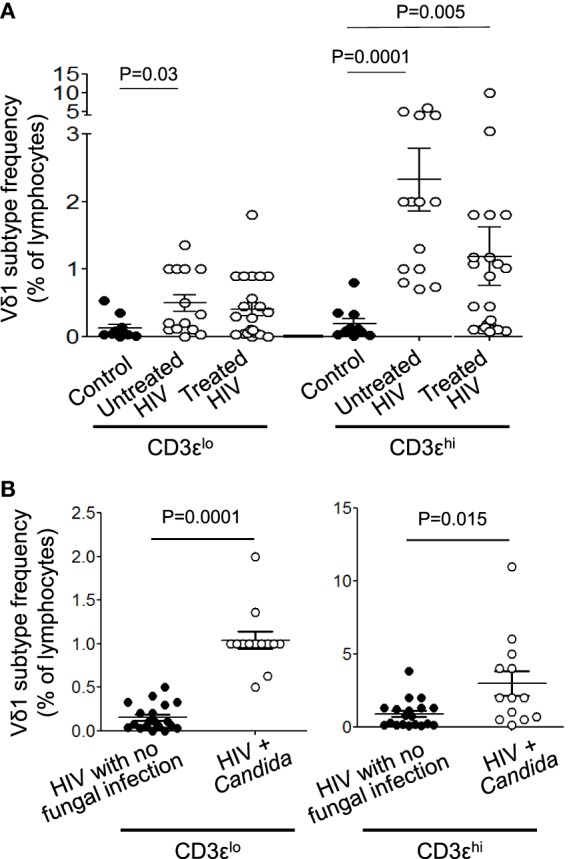
Both CD3ε^lo^ and CD3ε^hi^ Vδ1 T cells are expanded in untreated patients with human immunodeficiency virus (HIV) infection and especially in patients with *Candida* co-infection. Peripheral blood mononuclear cells were prepared from blood samples of 14 patients with untreated HIV infection, 22 patients receiving antiretroviral therapy and 23 healthy donors, stained with monoclonal antibodies specific for CD3ε and Vδ1 and analyzed by flow cytometry. **(A)** Scatter plots showing the frequencies of circulating CD3ε^lo^ and CD3ε^hi^ Vδ1 T cells in untreated and treated patients with HIV infection and healthy control subjects. **(B)** Scatter plots showing the frequencies of circulating CD3ε^lo^ and CD3ε^hi^ Vδ1 T cells in patients with HIV divided according to the absence (*n* = 19) or presence (*n* = 13) of *Candida albicans* co-infection. Groups were compared using the Mann–Whitney *U* test.

We next investigated if the percentage frequencies of CD3ε^lo^ and CD3ε^hi^ Vδ1 T cells correlated with the presence of HIV infection in untreated and ART-treated patients. PBMCs were prepared from 14 patients with untreated HIV infection, 22 patients receiving ART and 23 healthy donors, stained with mAbs specific for CD3ε and Vδ1 and analyzed by flow cytometry. Figure 4A shows that both subsets of Vδ1 T cells are expanded in the untreated patients, whereas CD3ε^hi^ Vδ1 T cells, only, are expanded in treated patients. There were no significant differences in the frequencies of CD3ε^lo^ and CD3ε^hi^ Vδ1 T cells in patients with HIV. We previously reported that Vδ1 T cells expand and release IL-17 in response to *C. albicans*, a common co-infection in patients with HIV (5). Figure 4B shows that the frequencies of both subsets of Vδ1 T cells were significantly higher in patients with *Candida* co-infection (*n* = 13) compared to patients with no evidence of fungal infection (*n* = 19), indicating that fungal infection makes a significant contribution to the increased frequencies of Vδ1 T cells reported in patients with HIV infection (28–33).

### CD3ε Expression by Vδ1 T Cells Can Be Modulated by Activation

**Figure 5:**
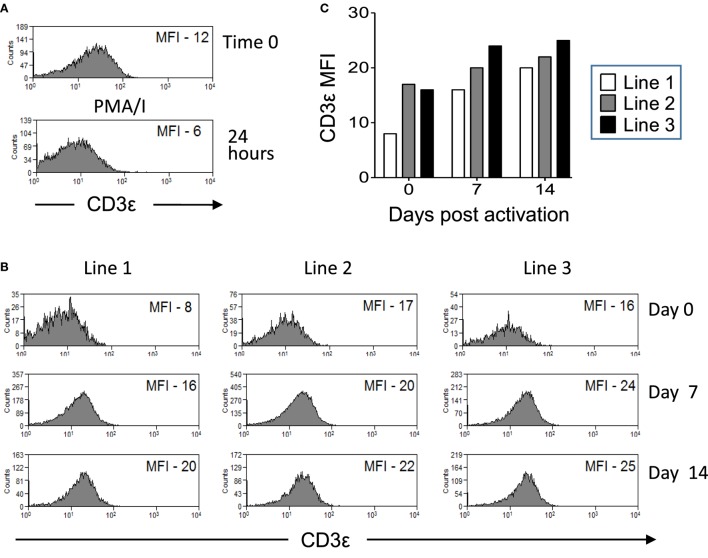
CD3ε expression by Vδ1 T cells is modulated over time post activation. CD3ε^hi^ and CD3ε^lo^ Vδ1 T cells were sorted from lines of expanded Vδ1 T cells from three donors. Cells were restimulated with phorbol myristate acetate with ionomycin (PMA/I) or heat-killed *Candida albicans* presented by monocyte-derived dendritic cell and PHA and cultured in the presence of interleukin (IL)-2. The expression of CD3ε by gated Vδ1 T cells was examined at times 0, 1, 7, and 14 days by flow cytometry. **(A)** Flow cytometry histograms showing downregulation of CD3ε by sorted CD3ε^hi^ Vδ1 T cells stimulated for 24 h with PMA/I. **(B)** Flow cytometry histograms showing the upregulation of CD3ε expression by sorted CD3ε^lo^ Vδ1 T cells after stimulation with *C. albicans* and culture with IL-2. **(C)** Mean fluorescence intensities (MFIs) of CD3ε expression by Vδ1 T cells from three donors over time after *C. albicans* stimulation.

We next investigated if CD3ε expression by Vδ1 T cells is stable or if it can be modulated by activation. CD3ε^hi^ and CD3ε^lo^ Vδ1 T cells were sorted from lines of Vδ1 T cells that were expanded from three donors. Cells were restimulated with PMA/I (Figure 5A) or DC pulsed with heat-killed *C. albicans* and PHA (Figure 5B) and cultured in the presence of IL-2. The expression of CD3ε by gated Vδ1 T cells was examined at times 0, 1, 7, and 14 days by flow cytometry. CD3ε expression by sorted CD3ε^hi^ Vδ1 T cells was transiently downregulated by activation with PMA/I (Figure 5A). Figures 5B,C show that CD3ε expression by CD3ε^lo^ Vδ1 T cells can be upregulated over time post reactivation with antigen. Thus, CD3ε can be transiently downregulated in CD3ε^hi^ Vδ1 T cells and upregulated in the CD3ε^lo^ population following activation, indicating that CD3ε expression is not fixed and that its downregulation is reversible.

### CD3ε^lo^ Vδ1 T Cells More Frequently Have TD Phenotypes Than CD3ε^hi^ Vδ1 T Cells

**Figure 6:**
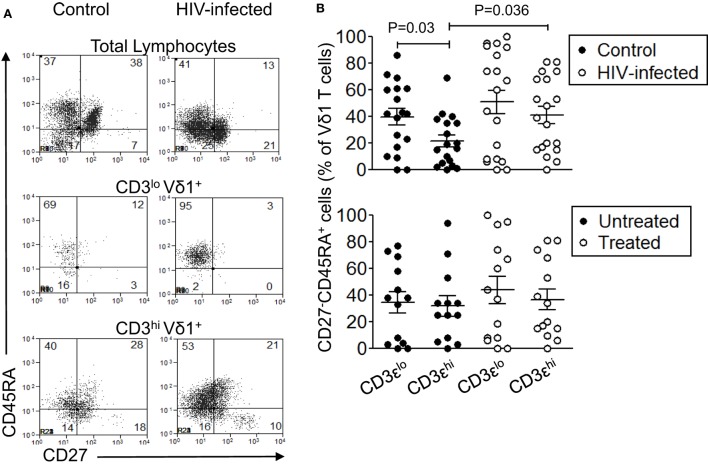
Significant proportions of CD3ε^lo^ Vδ1 T cells have terminally differentiated (TD) phenotypes. Peripheral blood mononuclear cell (PBMC) from 19 patients with human immunodeficiency virus (HIV) infection and 18 control subjects were enriched for γδ T cells using magnetic bead separation, stained with monoclonal antibodies specific for CD3ε, Vδ1, CD45RA, and CD27, and analyzed by flow cytometry. **(A)** Representative flow cytometry dot plots showing CD45RA and CD27 expression by total lymphocytes (upper panels), CD3ε^lo^ Vδ1 T cells (center panels), and CD3ε^hi^ Vδ1 T cells (bottom panels) in a control subject (left panels) and a patient with HIV infection (right panels). **(B)** Scatter plots showing the frequencies of CD3ε^lo^ and CD3ε^hi^ Vδ1 T cells from the patients and controls (upper graph) that expressed TD (CD45RA^+^CD27^−^) phenotypes. The lower graph shows the frequencies of CD3ε^lo^ and CD3ε^hi^ Vδ1 T cells from untreated (*n* = 13) and antiretroviral therapy-treated (*n* = 14) patients who expressed TD phenotypes. Groups were compared using the Mann–Whitney *U* test.

The differentiation status of CD3ε^lo^ and CD3ε^hi^ Vδ1 T cells in 19 patients with HIV infection and 18 control subjects was examined by flow cytometric analysis of CD45RA and CD27 co-expression (34, 35). Figure 6A shows that significant proportions of total lymphocytes and gated CD3ε^hi^ Vδ1 T cells within γδ T cell-enriched PBMC from patients and controls expressed naïve (CD45RA^+^CD27^+^), central memory (CD45RA^−^CD27^+^), effector memory (CD45RA^−^CD27^−^), and TD (CD45RA^+^CD27^−^) phenotypes. By contrast, CD3ε^lo^ Vδ1 T cells from control subjects exhibited significantly higher frequencies of TD cells compared to CD3ε^hi^ Vδ1 T cells (Figures 6A,B). A similar increase in TD cells among CD3ε^lo^ Vδ1 T cells was found in the patients with HIV, with 90–100% of these cells being CD45RA^+^CD27^+^ in some patients, but this did not reach statistical significance. Interestingly, the proportions of CD3ε^hi^ Vδ1 T cells that expressed TD phenotypes were higher in the HIV patients compared to control subjects. When the HIV-infected patients were divided into untreated (*n* = 13) and ART-treated (*n* = 14) subjects, the proportions of CD3ε^lo^ Vδ1 T cells expressing TD phenotypes was only marginally higher than those of CD3ε^hi^ Vδ1 T cells (Figure 6B). These results show that significant proportions of CD3ε^lo^ Vδ1 T cells express TD phenotypes, suggesting that they are exhausted as a result of HIV infection.

### CD3ε^lo^ Vδ1 T Cells More Frequently Express PD-1, but Not LAG-3 or CD31, Than CD3ε^hi^ Vδ1 T Cells

**Figure 7:**
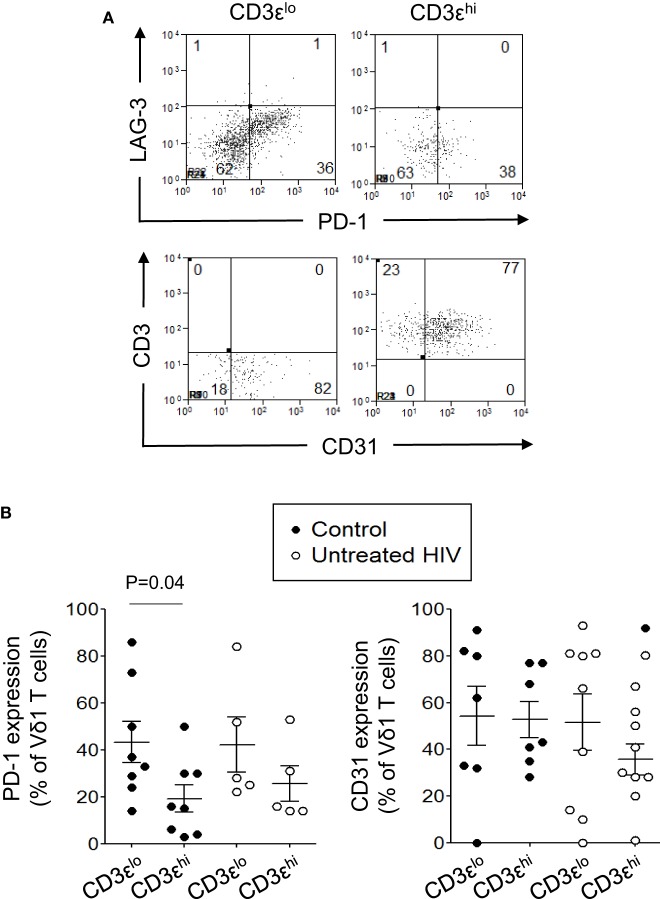
CD3ε^lo^ Vδ1 T cells more frequently express programmed death-1 (PD-1), but not lymphocyte-activation gene 3 (LAG-3) or CD31, than CD3ε^hi^ Vδ1 T cells. Peripheral blood mononuclear cell prepared from eight healthy donors and five untreated patients with human immunodeficiency virus (HIV) were enriched for γδ T cells using magnetic bead separation, stained with monoclonal antibodies specific for CD3ε, Vδ1, PD-1, LAG-3, and CD31 and analyzed by flow cytometry. **(A)** Flow cytometry dot plots showing PD-1, LAG-3, and CD31 expression by gated CD3ε^lo^ and CD3ε^hi^ Vδ1 T cells from a patient with HIV. **(B)** Scatter plots showing the frequencies of CD3ε^lo^ and CD3ε^hi^ Vδ1 T cells from the patients with HIV and control subjects that expressed PD-1 and CD31. Groups were compared using the Mann–Whitney *U* test.

Human immunodeficiency virus can induce the expression of the inhibitory receptors PD-1 and LAG-3 on HIV-specific T cells leading to their inactivation (36–42). Since Vδ1 T cells with TD phenotypes are preserved in patients with HIV infection, we investigated if CD3ε^lo^ and CD3ε^hi^ Vδ1 T cells from five untreated patients with HIV infection and eight control subjects express PD-1 or LAG-3. We also investigated if these cells express the naïve T cell marker CD31 (43). Figure 7 shows that PD-1 is expressed at higher levels on CD3ε^lo^ Vδ1 T cells compared to CD3ε^hi^ Vδ1 T cells from eight healthy donors. A similar trend, although not statistically significant was found in five untreated HIV patients (Figure 7B). PD-1 expression by CD3ε^lo^ and CD3ε^hi^ Vδ1 T cells was similar in patients and control subjects. By contrast, neither CD3ε^lo^ nor CD3ε^hi^ Vδ1 T cells from patients or controls expressed LAG-3. CD31 was expressed by variable proportions of CD3ε^lo^ and CD3ε^hi^ Vδ1 T cells and its expression was not altered in patients with HIV (Figure 7).

### CD3ε^lo^ Vδ1 T Cells Exhibit Impaired IL-17 Production

**Figure 8:**
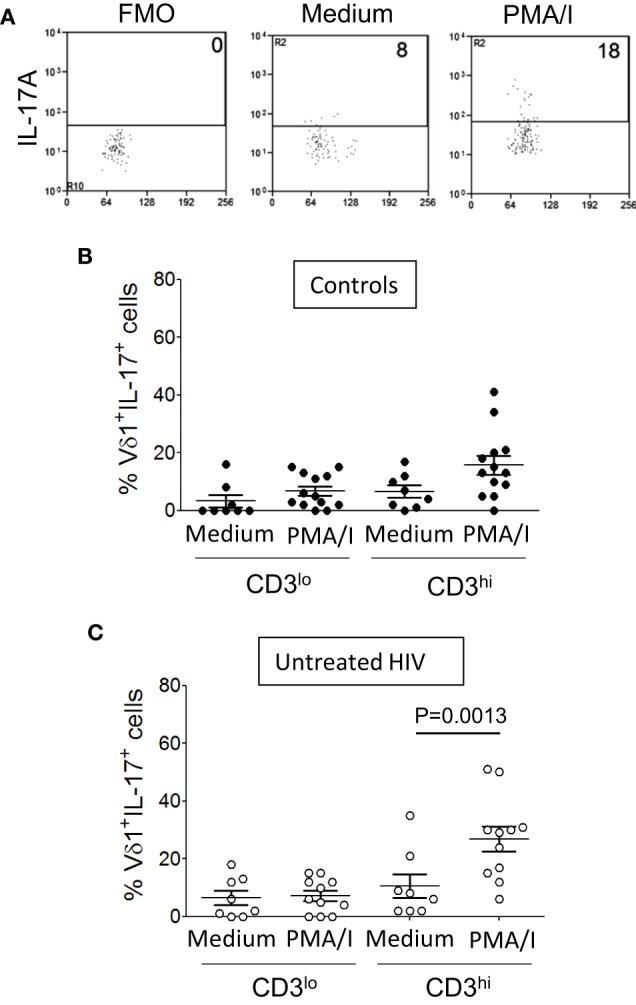
CD3ε^lo^ Vδ1 T cells exhibit impaired interleukin (IL)-17 production. Peripheral blood mononuclear cell (PBMC) from 13 healthy donors and 11 patients with untreated human immunodeficiency virus (HIV) infection were enriched for γδ T cells using magnetic bead separation. The cells were stimulated for 6 h with phorbol myristate acetate with ionomycin (PMA/I) or incubated in medium alone in the presence of brefeldin A. **(A)** Flow cytometry dot plots showing IL-17A expression by gated CD3ε^hi^ Vδ1 T cells within unstimulated PBMC (center panel) and PMA/ionomycin-stimulated PBMC (right panel) from a patient with HIV. The left panel shows a fluorescence minus one (FMO) control dot plot. **(B,C)** Scatter plots show the frequencies of CD3ε^lo^ and CD3ε^hi^ Vδ1 T cells from healthy donors **(B)** and HIV patients **(C)** that produced IL-17. Groups were compared using the Mann–Whitney *U* test.

The increased expression of PD-1 and TD phenotypes of CD3ε^lo^ Vδ1 T cells suggest that these cells are in a state of exhaustion. We and others have shown that Vδ1 T cells are rapid and potent producers of IL-17 (4, 5). We investigated if CD3ε^lo^ and CD3ε^hi^ Vδ1 T cells from patients with HIV infection and control subjects differ in their ability to produce IL-17. γδ T cell-enriched PBMC from 13 healthy donors and 11 patients with HIV were stimulated for 6 h with PMA/I or incubated in medium alone and IL-17A expression by gated CD3ε^lo^ and CD3ε^hi^ Vδ1 T cells was examined by flow cytometry (Figure 8A). Figures 8B,C show that PMA/I treatment induced the production of IL-17 by significant numbers of CD3ε^hi^ Vδ1 T cells from both control subjects and HIV patients. However, stimulation of CD3ε^lo^ Vδ1 T cells with PMA/I did not lead to IL-17 production, suggesting that these cells are at least partially inactivated (Figure 8).

## Discussion

Numerous studies have shown that Vδ1 T cells are proportionally expanded in patients with HIV (28–33). Vδ1 T cells may contribute to immunity against HIV by killing infected CD4^+^ T cells (21, 25), releasing antiviral cytokines (4, 25, 27) and chemokines (23). They may also contribute to the immunodeficiency associated with HIV infection, by depleting CD4^+^ T cells (26). In this study, we have shown that Vδ1 T cells are not expanded in our patients with HIV infection, but their overall percentages are increased, suggesting that these cells are merely preserved in patients with HIV, while other cells are depleted. Since Vδ1 T cells are an important source of innate IL-17 (4, 5), it is also possible that their main role in patients with HIV is to stimulate immunity against co-infecting bacteria and fungi (1–5). Consistent with this hypothesis, we and others have found that Vδ1 T cells expand and produce IL-17 in response to *C. albicans* and that their frequencies are highest in HIV-positive patients with *Candida* co-infection (4, 5). Vδ1 T cells are also thought to be major producers of IL-17 in patients with colorectal cancer, in whom they have reduced IFN-γ production (44, 45). However, very few of these cells from healthy donors and patients with primary immunodeficiencies were reported to produce IL-17 (46), suggesting that IL-17 production by Vδ1 T cells is dependent on environmental factors, such as infection.

The TCR consists of a clonotypic αβ or a γδ glycoprotein heterodimer, generated by somatic recombination of germline gene segments, that recognizes antigens associated with antigen-presenting molecules, such as MHC, MR1, or CD1 (10). The TCR polypeptides associate with the CD3 complex, formed by the CD3 γ, δ (not to be confused with the TCR γ and δ polypeptides), ε and ζ subunits, which are invariable and mediate signal transduction. CD3ε can form heterodimers with CD3γ and CD3δ, while CD3ζ frequently exists as a homodimer, and CD3δε, CD3γε, and CD3ζζ are all capable of transducing activating signals in response to TCR ligation (9, 10). The CD3 γ, δ, ε, and ζ polypeptides all contain ITAMs in their cytoplasmic domains, which are required for intracellular assembly and surface expression of the TCR and signal transduction events that mediate thymocyte maturation and mature αβ T cell activation (47–50). Humans and mice lacking CD3ε have no αβ or γδ T cells (49, 51), indicating an absolute requirement for CD3ε in early T cell development. However, unlike in αβ T cells, γδ TCR rearrangement can occur in the absence of CD3ε (50) and some mature γδ T cells do not express CD3ε (52). γδ TCRs can also signal through FcRγ homodimers and CD3ζ-FcRγ heterodimers (52).

In this study, we have identified two populations of Vδ1 T cells, one of which expresses normal levels of CD3ε and the other which appears to express no or low levels of CD3ε, but normal levels of CD3ζ. CD3ε^lo^ and CD3ε^hi^ Vδ1 T cells were present in PBMC from patients with HIV and in control subjects and in expanded lines of Vδ1 T cells. Using confocal microscopy of sorted CD3ε^lo^ and CD3ε^hi^ Vδ1 T cells, we show that the absence of CD3ε is unlikely to be due to internalization of the polypeptide, since intracellular CD3ε was not detected. To investigate the stability of CD3ε expression, CD3ε^lo^ and CD3ε^hi^ Vδ1 T cells were sorted from lines of Vδ1 T cells and restimulated with *C. albicans* and cultured in the presence of IL-2. We found that CD3ε^hi^ Vδ1 T cells could downregulate CD3ε and CD3ε^lo^ Vδ1 T cells could upregulate CD3ε expression, suggesting that the expression of this component of CD3 can be modulated by activation and that its downregulation is reversible. CD3ε expression is required for progression of thymocyte maturation from the double positive CD4^+^CD8^+^ stage to the single positive CD4^+^ or CD8^+^ stage and for assembly of the pre-TCR (49, 50, 53, 54), but appears to be dispensible in mature T cells, where it may act to amplify weak signals from the TCR (55, 56). Thus, it is possible that CD3ε^lo^ Vδ1 T cells have a lower responsiveness to antigenic stimulation than CD3ε^hi^ Vδ1 T cells. Interestingly, Vδ1 TCR expression was slightly lower in CD3ε^lo^ compared to CD3ε^hi^ Vδ1 T cells in HIV patients and control subjects, adding further support to this idea. CD3ε contains endocytosis determinants that may contribute to the up- and downregulation of CD3ε on T cells (57) and recent studies have provided evidence that CD3ε expression can be downregulated by tumor-educated tolerogenic DC (58) and possibly by HIV (59, 60). We found that both CD3ε^lo^ and CD3ε^hi^ Vδ1 T cells are expanded in patients with untreated HIV infection compared to control subjects, but especially in patients with *C. albicans* co-infection. Thus, CD3ε^lo^ Vδ1 T cells accounted for 0.1% of lymphocytes in controls, compared to 0.5% in untreated HIV patients (*P* = 0.03) and >1% in patients with HIV and *Candida* infection (*P* = 0.0001). Likewise, CD3ε^hi^ Vδ1 T cells accounted for 0.2% of controls, compared to 2.3% of untreated HIV patients (*P* = 0.0001) and >3% of patients with HIV and *Candida* infection (*P* = 0.015). Future studies are required to identify the antigenic specificities of the Vδ1 TCR and to ascertain if Vδ1 T cell numbers or the ratios of CD3ε^hi^ to CD3ε^lo^ Vδ1 T cells can be used as a prognostic marker of *Candida* co-infection.

To determine if CD3ε^lo^ Vδ1 T cells display phenotypic or functional differences from CD3ε^hi^ Vδ1 T cells, PBMC freshly isolated from healthy donors were enriched for γδ T cells and further analyzed by flow cytometry. We found that CD3ε^lo^ Vδ1 T cells more frequently have TD phenotypes and express PD-1, but not LAG-3, compared to CD3ε^hi^ Vδ1 T cells, suggesting that they have previously been activated and exist in a state of inactivation. PD-1 and LAG-3 expression by HIV-specific CD4^+^ and CD8^+^ T cells is a feature of HIV infection, is associated with T-cell exhaustion and disease progression, and is thought to promote viral persistence (36–42). Our finding that Vδ1 T cells, and especially the CD3ε^lo^ subset of Vδ1 T cells, frequently express PD-1 indicates that this induction of exhaustion in HIV infection extends to γδ T cells and suggests that mAb blocking of PD-1 may benefit patients with HIV (61). Previous workers have reported a skewing of Vγ9Vδ2 T cells toward TD in patients with HIV (32, 62), which is associated with impaired IFN-γ production (63). We tested if CD3ε^lo^ Vδ1 T cells display properties of exhaustion by testing their ability to produce IL-17, a cardinal function of Vδ1 T cells (4, 5). We found that significant proportions of CD3ε^hi^ Vδ1 T cells, but not CD3ε^lo^ Vδ1 T cells, produced IL-17 in response to PMA/I stimulation *ex vivo*. Therefore, CD3ε^lo^ Vδ1 T cells may represent a population of inactive, TD T cells. Since IL-17 production is only one of multiple effector activities of Vδ1 T cells, future studies are required to determine if other activities, such as IFN-γ production, are deficient in CD3ε^lo^ Vδ1 T cells. Vδ1 and Vγ9Vδ2 T cells expressing low levels of CD3 and exhibiting impaired responses to stimulation have been reported to accumulate in sites of active *Mycobacterium tuberculosis* infection (64, 65) and Paget et al. (66) reported that murine Vγ6Vδ1^+^ T cells with low levels of CD3 predominantly produce IFN-γ whereas the same cells with high levels of CD3 produce IL-17. Thus, modulation of CD3ε expression may be a general mechanism for the regulation of γδ T cell activity.

The results of this study indicate that Vδ1 T cells persist in the blood of patients with untreated HIV infection, and especially in patients with *Candida* co-infection, while other T cells are depleted. Although it is not known if Vδ1 T cells can directly recognize HIV or HIV-infected cells, a recent study has shown that γδ TCR exposure to viruses can promote the expansion of virus-reactive T cells, providing strong evidence that γδ T cells mediate adaptive immune responses to viruses (67). Previous studies have demonstrated that Vδ1 T cells proliferate and release IL-17 in response to *C. albicans*, by a mechanism that requires IL-23 release from DC (4, 5). The preservation of Vδ1 T cells in patients whose IL-17-producing CD4^+^ T cells may be depleted by HIV, identifies Vδ1 T cells as an alternative potential source of IL-17. However, it appears that significant proportions of Vδ1 T cells in patients with HIV have been driven to a state of inactivation, expressing TD phenotypes and the inhibitory receptor PD-1 and failing to produce IL-17 upon stimulation. Downregulation of CD3ε, a signaling molecule known to augment TCR-mediated responses (55, 56), may represent another mechanism by which the effector functions of Vδ1 T cells can be inhibited.

## Ethics Statement

Ethical approval for this study was obtained from the Joint Research Ethics Committee of St. James’s Hospital and Tallaght Hospitals, Dublin, and all participants gave written, informed consent.

## Author Contributions

PD, TR, and DD: conceived the study. PD, CM, MF, KD, AP, JO, A Long, and MD: performed experiments, acquired and analyzed data. DR, SO, and FM: directed and coordinated sample collection. A Loy, JW, and FM: performed sample collection. DD: wrote the manuscript.

## Conflict of Interest Statement

The authors declare that the research was conducted in the absence of any commercial or financial relationships that could be construed as a potential conflict of interest.

## Abbreviations

ART, antiretroviral therapy; DC, dendritic cell; FITC, fluorescein isothiocyanate; FMO, fluorescence minus one; HIV, human immunodeficiency virus; IL-17, interleukin-17; IFN-γ, interferon-γ; mAb, monoclonal antibody; MFI, mean fluorescence intensity; MHC, major histocompatibility complex; PBMCs, peripheral blood mononuclear cells; PBS, phosphate buffered saline; PD-1, programmed death-1; PMA/I, phorbol myristate acetate with ionomycin; TCR, T cell receptor; TD, terminally differentiated.
